# Microbiota-derived Trimethylamine N-oxide Predicts Cardiovascular Risk After STEMI

**DOI:** 10.1038/s41598-019-48246-6

**Published:** 2019-08-12

**Authors:** Yasushi Matsuzawa, Hidefumi Nakahashi, Masaaki Konishi, Ryosuke Sato, Chika Kawashima, Shinnosuke Kikuchi, Eiichi Akiyama, Noriaki Iwahashi, Nobuhiko Maejima, Kozo Okada, Toshiaki Ebina, Kiyoshi Hibi, Masami Kosuge, Tomoaki Ishigami, Kouichi Tamura, Kazuo Kimura

**Affiliations:** 1 Division of Cardiology, Yokohama City University Medical Center, Yokohama, Japan; 2 Department of Medical Science and Cardiorenal Medicine, Yokohama City University Graduate School of Medicine, Yokohama, Japan

**Keywords:** Cardiology, Medical research, Myocardial infarction

## Abstract

Trimethylamine N-oxide (TMAO), a metabolite derived from the gut microbiota, is proatherogenic and associated with cardiovascular events. However, the change in TMAO with secondary prevention therapies for ST-segment elevation acute myocardial infarction (STEMI) remains unclear. The purpose of this study was to investigate the sequential change in TMAO levels in response to the current secondary prevention therapies in patients with STEMI and the clinical impact of TMAO levels on cardiovascular events We included 112 STEMI patients and measured plasma TMAO levels at the onset of STEMI and 10 months later (chronic phase). After the chronic-phase assessment, patients were followed up for cardiovascular events. Plasma TMAO levels significantly increased from the acute phase to the chronic phase of STEMI (median: 5.63 to 6.76 μM, P = 0.048). During a median period of 5.4 years, 17 patients experienced events. The chronic-phase TMAO level independently predicted future cardiovascular events (adjusted hazard ratio for 0.1 increase in log chronic-phase TMAO level: 1.343, 95% confidence interval 1.122–1.636, P = 0.001), but the acute-phase TMAO level did not. This study demonstrated the clinical importance of the chronic-phase TMAO levels on future cardiovascular events in patients after STEMI.

## Introduction

The long-term prognosis of patients after myocardial infarction has been considerably improved; this improvement has been achieved by developing treatments for traditional risk factors of atherosclerosis, such as hypertension, dyslipidemia, and diabetes. However, atherosclerosis is a complex vascular inflammatory disease, and it has been suggested that most of the mechanisms of atherosclerosis are unknown^1^. The residual risk in patients after ST-segment elevation acute myocardial infarction (STEMI) is still high for second cardiovascular events^2^.

Recently, the microbiota has been suggested to be linked to many inflammatory conditions and immunological disorders^3^, and much interest has been focused on the gut microbiota’s role in metabolic disorders and cardiovascular diseases. Several studies have reported that trimethylamine N-oxide (TMAO), one of the metabolites derived from the gut flora, may play an important role in cardiovascular disease progression^4–7^. In 2013, Tang *et al*. reported a strong relationship between elevated systemic TMAO levels and an increased risk of incident major adverse cardiovascular events in 4,007 stable patients undergoing elective diagnostic cardiac evaluations^5^. This seminal study laid the foundation for the identification of an emerging gut-flora-dependent metabolite and inspired subsequent studies aiming to uncover a possible novel mechanism of cardiovascular disease and to translate it into clinical practice. In 2016, a study with animal models demonstrated that TMAO promotes vascular inflammation, inducing aortic endothelial activation and upregulation of adhesion proteins^8^. Another study *in vivo* reported that gut microbiota modulates platelet hyperresponsiveness and clot formation by generating TMAO^7^.

Dietary interventions can modify the composition of the microbiotas^9,10^. However, the sequential change in TMAO levels by the current secondary prevention strategies (including statins, diet, and exercise therapy) in patients after STEMI has not been reported. Furthermore, the clinical impact of TMAO before and after secondary preventive therapies in STEMI patients on the progression of atherosclerosis and subsequent cardiovascular events is still unknown.

We hypothesized that TMAO levels change after secondary preventive therapies for STEMI and that chronic-phase TMAO levels are more important than acute-phase levels in preventing secondary cardiovascular events. Thus, the first aim of the current study was to assess sequential changes in TMAO levels between the acute phase of STEMI and 10 months after the implementation of secondary preventive treatments. The second aim was to compare the impact of the acute-phase and chronic-phase TMAO levels on coronary plaque progression and cardiovascular events in patients after STEMI.

## Results

A total of 112 STEMI patients were included. The median age at the onset of STEMI was 63 years, and 88% were male (Table 1). All patients were instructed on lifestyle modifications and were prescribed medications for secondary prevention. After 10 months, body weight tended to be decreased (median value: from 66.3 to 65.3 kg, P = 0.33), and median values for hemoglobin A1c (HbA1c), low-density lipoprotein (LDL) cholesterol, high-density lipoprotein (HDL) cholesterol, and triglycerides changed from 6.0 to 6.0% (P = 0.52), 140 to 81 mg/dl (P < 0.001), 46 to 46 mg/dl (P = 0.41), and 114 to 133 mg/dl (P = 0.07), respectively (Tables 1 and 2). As determined by parametric methods, the mean value of HbA1c was significantly decreased from 6.5 to 6.3%, P = 0.037. In particular, among the patients with HbA1c ≥ 7.0% at baseline, the mean HbA1c levels decreased from 8.5 to 7.1% (P < 0.001). Aspirin was prescribed for all patients, statins for 98%, β-receptor blockers for 86%, and angiotensin-converting enzyme inhibitors or angiotensin II receptor blockers for 96% (Table 2). These patients underwent coronary angiography at 10 months after STEMI and were thereafter followed for cardiovascular events. Over a median period of 5.4 years (interquartile range, 4.2 to 6.9 years), we observed five deaths, five cases of nonfatal acute myocardial infarction, two cases of unstable angina pectoris requiring revascularization, and five cases of nonfatal stroke. Within 10 months after STEMI, only 1 event (acute myocardial infarction due to coronary spasm) occurred, and it was not counted for analysis in this study.

**Table 1:** Baseline characteristics at the onset of STEMI according to the acute-phase plasma TMAO levels.

	All patients N = 112	TMAO levels in the acute phase		P
		Below median n = 57	Above median n = 55	
Age, years	63 (56–71)	60 (45–70)	65 (61–72)	0.009
Male sex, n (%)	99 (88%)	49 (86%)	50 (91%)	0.56
Height, cm	165 (162–170)	167 (162–172)	165 (160–169)	0.063
Body weight, kg	66.3 (59.0–75.5)	68.4 (59.4–76.8)	64.6 (58.0–74.1)	0.34
Body mass index, kg/m^2^	24.1 (22.0–26.6)	23.7 (21.8–27.3)	24.2 (22.1–25.8)	0.85
Systolic blood pressure, mmHg	140 (120–158)	142 (118–157)	135 (120–158)	0.85
Diastolic blood pressure, mmHg	79 (62–93)	76 (61–94)	80 (66–91)	0.81
Coronary risk factors				
Hypertension, n (%)	54 (48%)	24 (42%)	30 (55%)	0.26
Dyslipidemia, n (%)	69 (62%)	42 (74%)	27 (50%)	0.012
Diabetes, n (%)	32 (29%)	19 (33%)	13 (24%)	0.30
Smoking, n (%)	83 (74%)	42 (74%)	41 (75%)	>0.99
Laboratory data on admission				
Total cholesterol, mg/dl	212 (182–233)	212 (192–235)	206 (177–230)	0.30
HDL cholesterol, mg/dl	46 (39–57)	45 (38–56)	47 (40–58)	0.43
LDL cholesterol, mg/dl	140 (121–157)	143 (123–156)	133 (112–159)	0.20
Triglycerides, mg/dl	114 (66–191)	107 (64–203)	120 (74–189)	0.87
Glucose, mg/dl	166 (137–201)	170 (141–206)	153 (135–194)	0.26
HbA1c, %	6.0 (5.7–6.7)	6.2 (5.7–7.2)	5.9 (5.7–6.1)	0.11
eGFR, ml/min/1.73 m^2^	69 (59–81)	72 (62–90)	69 (58–79)	0.11
BNP, pg/ml	25 (10–55)	22 (10–56)	29 (11–55)	0.48
CRP, mg/L	1.3 (0.6–2.6)	1.3 (0.6–2.5)	1.3 (0.6–2.6)	0.87
Anterior wall MI, n (%)	55 (49%)	27 (47%)	28 (51%)	0.85
Peak CK-MB, IU/L	195 (94–319)	179 (89–299)	205 (109–325)	0.21
Triple vessel disease, n (%)	18 (16%)	11 (19%)	7 (13%)	0.34
Initial SYNTAX score	22 (16–27)	22 (17–26)	23 (14–27)	0.67
Stent type				0.92
Bare metal stents, n (%)	105 (94%)	53 (93%)	52 (95%)	
Drug eluting stents, n (%)	2 (2%)	1 (2%)	1 (2%)	
TMAO levels in the chronic phase, μM	6.76 (3.82–12.53)	6.67 (3.59–11.65)	7.19 (4.99–15.60)	0.108
Medications at the onset of STEMI				
Aspirin, n (%)	5 (5%)	3 (5%)	2 (4%)	> 0.99
Beta blocker, n (%)	5 (4%)	1 (2%)	4 (7%)	0.20
ACE-I/ARB, n (%)	24 (21%)	14 (25%)	10 (18%)	0.49
Statin, n (%)	17 (15%)	9 (16%)	8 (15%)	> 0.99

Data are shown as median (first and third quartile) or number (%).

ACE-I: angiotensin-converting enzyme inhibitor; ARB: angiotensin II receptor blocker; BNP: B-type natriuretic peptide; CK-MB: creatine kinase-myocardial band; CRP: C-reactive protein; eGFR: estimated glomerular filtration rate; HDL: high-density lipoprotein; LDL: low-density lipoprotein; MI: myocardial infarction; STEMI: ST-segment elevation acute myocardial infarction; TMAO: trimethylamine N-oxide.

**Table 2:** Patient characteristics at 10 months after onset of STEMI according to chronic-phase plasma TMAO levels.

	All patients N = 112	TMAO levels in the chronic phase		P
		Below median n = 56	Above median n = 56	
Age, years	64 (57–72)	62 (53–69)	66 (59–73)	0.062
Male sex, n (%)	99 (88%)	54 (96%)	45 (80%)	0.016
Body weight, kg	65.3 (58.5–73.8)	68.1 (60.0–77.0)	62.5 (53.6–71.1)	0.016
Body mass index, kg/m^2^	24.1 (22.0–26.6)	24.6 (22.5–26.1)	23.4 (20.5–25.5)	0.033
Systolic blood pressure, mmHg	117 (108–132)	116 (108–130)	118 (110–134)	0.50
Diastolic blood pressure, mmHg	70 (61–80)	70 (62–82)	68 (60–78)	0.49
Coronary risk factors				
Hypertension, n (%)	54 (48%)	26 (46%)	28 (50%)	0.85
Dyslipidemia, n (%)	69 (62%)	34 (62%)	35 (63%)	>0.99
Diabetes, n (%)	32 (29%)	17 (30%)	15 (27%)	0.83
Smoking, n (%)	83 (74%)	40 (71%)	43 (77%)	0.67
Laboratory data after 10 months				
Total cholesterol, mg/dl	153 (138–167)	153 (137–174)	153 (139–161)	0.50
HDL cholesterol, mg/dl	46 (39–55)	44 (40–53)	46 (37–59)	0.72
LDL cholesterol, mg/dl	81 (70–96)	85 (76–100)	79 (69–92)	0.042
Triglycerides, mg/dl	133 (97–204)	132 (99–213)	138 (83–199)	0.59
Glucose, mg/dl	118 (100–146)	117 (100–135)	124 (101–157)	0.26
HbA1c, %	6.0 (5.8–6.6)	6.0 (5.8–6.7)	6.1 (5.8–6.5)	0.71
eGFR, ml/min/1.73 m^2^	69 (56–78)	70 (59–80)	66 (56–75)	0.116
BNP, pg/ml	38 (15–57)	32 (15–57)	44 (17–60)	0.29
CRP, mg/L	0.69 (0.36–1.54)	0.66 (0.37–1.63)	0.75 (0.35–1.47)	0.79
Anterior wall MI, n (%)	55 (49%)	29 (52%)	26 (46%)	0.71
Peak CK-MB, IU/L	195 (94–319)	204 (89–324)	178 (96–284)	0.69
SYNTAX score 10 months later	11 (7–18)	10 (6–15)	11 (7–19)	0.11
TMAO levels in the acute phase, μM	5.63 (3.20–10.38)	5.21 (2.89–9.39)	5.89 (3.45–12.80)	0.53
Medications after 10 months				
Aspirin, n (%)	112 (100%)	56 (100%)	56 (100%)	>0.99
P2Y12 inhibitors, n (%)	109 (97%)	53 (95%)	56 (100%)	0.24
Clopidogrel, n (%)	64 (57%)	30 (54%)	33 (59%)	
Ticlopidine, n (%)	43 (38%)	22 (39%)	21 (38%)	
Prasugrel, n (%)	1 (1%)	0 (0%)	1 (2%)	
Beta blocker, n (%)	96 (86%)	51 (91%)	45 (80%)	0.18
ACE-I/ARB, n (%)	107 (96%)	51 (91%)	56 (100%)	0.057
Statin, n (%)	110 (98%)	56 (100%)	54 (96%)	0.50

Data are shown as median (first and third quartile) or number (%).

ACE-I: angiotensin-converting enzyme inhibitor, ARB: angiotensin II receptor blocker, BNP: B-type natriuretic peptide, CK-MB: creatine kinase-myocardial band, CRP: C-reactive protein, eGFR: estimated glomerular filtration rate, HDL: high-density lipoprotein, LDL: low-density lipoprotein, MI: myocardial infarction, and TMAO: trimethylamine N-oxide.

### Distribution of and change in plasma TMAO levels after the implementation of optimal secondary preventive therapies

**Figure 1:**
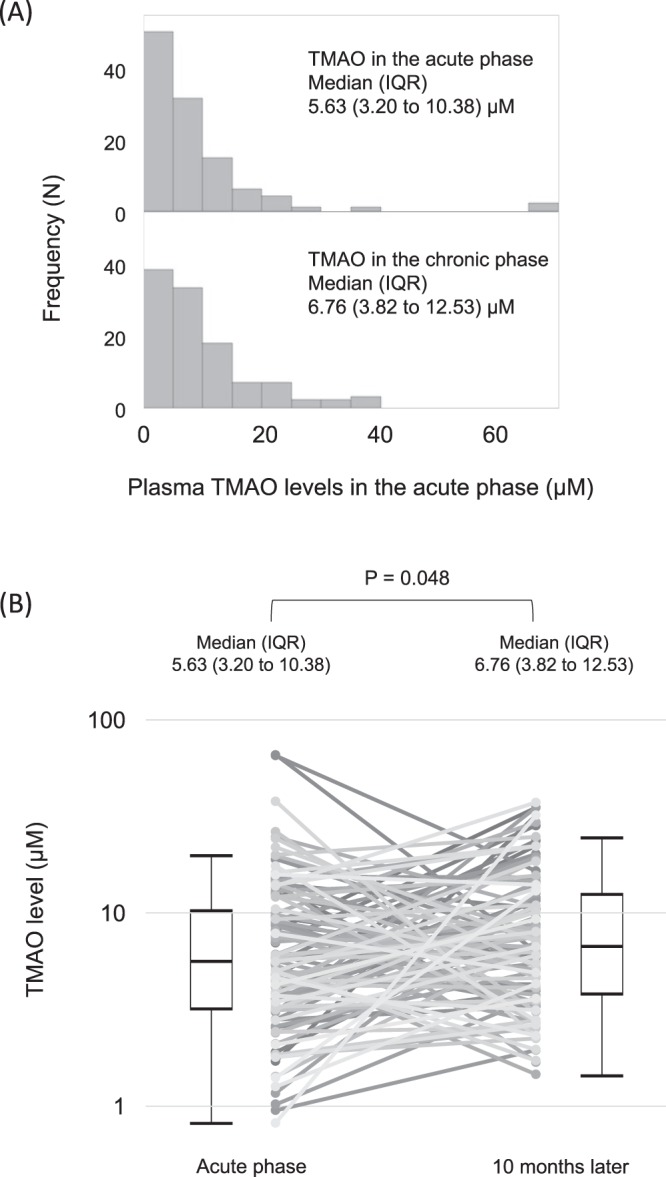
The distribution of TMAO and change after secondary prevention therapies. (**A**) The distribution of TMAO levels at the onset of STEMI and 10 months later. (**B**) Change in TMAO levels between the acute and chronic phases. IQR: interquartile range; STEMI: ST-segment elevation acute myocardial infarction; TMAO: trimethylamine N-oxide.

We created histograms of plasma TMAO levels in the acute and chronic phases (Fig. 1A), which show a markedly right-skewed distribution. The median value (25th to 75th percentile) of plasma TMAO levels in the acute phase was 5.63 μM (3.20 to 10.38), and the values ranged from 0.82 to 66 µM. The median plasma levels of TMAO significantly increased from the acute phase to the chronic phase of STEMI (from 5.63 to 6.76 μM, P = 0.048, Fig. 1B).

### Clinical characteristics according to TMAO levels

To show the clinical characteristics of the 112 STEMI patients, we created 2 tables according to the median value of plasma TMAO levels in the acute phase and the chronic phase (Tables 1 and 2, respectively). Age was significantly increased and the proportion of dyslipidemia was significantly decreased in the patients with high TMAO levels in the acute phase (Table 1). Similarly, the patients with high TMAO levels in the chronic phase tended to be older than those with lower TMAO (Table 2). The proportion of men and values of body weight, body mass index, and LDL cholesterol level were significantly lower in the patients with high TMAO levels in the chronic phase than in those with lower TMAO.

### Association between TMAO levels and SYNTAX scores

**Figure 2:**
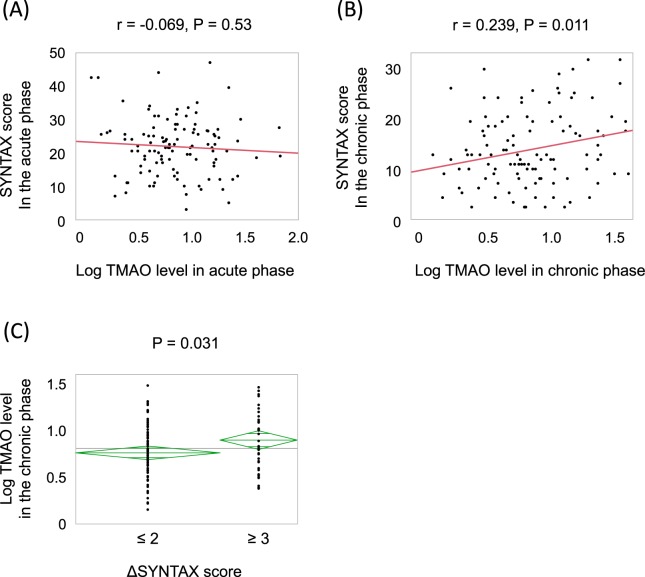
The association of TMAO levels with SYNTAX score. (**A**) The relationship between acute-phase TMAO levels and SYNTAX scores. (**B**) The relationship between TMAO levels and SYNTAX score in the chronic phase. (**C**) Chronic-phase TMAO levels and plaque progression. ΔSYNTAX score = (SYNTAX score in chronic phase) − (Residual SYNTAX score in acute phase).

Figure 2 demonstrates the association between plasma TMAO levels and coronary plaque SYNTAX scores. The acute-phase TMAO levels were not significantly associated with initial SYNTAX scores (r = −0.069, P = 0.53, Fig. 2A), whereas there was a significant positive relationship between chronic-phase TMAO levels and chronic-phase SYNTAX scores (r = 0.239, P = 0.011, Fig. 2B). To assess the impact of TMAO levels on plaque progression, we compared chronic-phase TMAO levels between patients with plaque progression (ΔSYNTAX score ≥ 3 [the highest tertile]) and those without. Chronic-phase TMAO levels were significantly higher in patients with plaque progression than in those without (log chronic TMAO levels: 0.950 ± 0.364 versus 0.805 ± 0.304, P = 0.031, Fig. 2C).

### Relationship between plasma TMAO levels and long-term cardiovascular outcomes after STEMI

**Figure 3:**
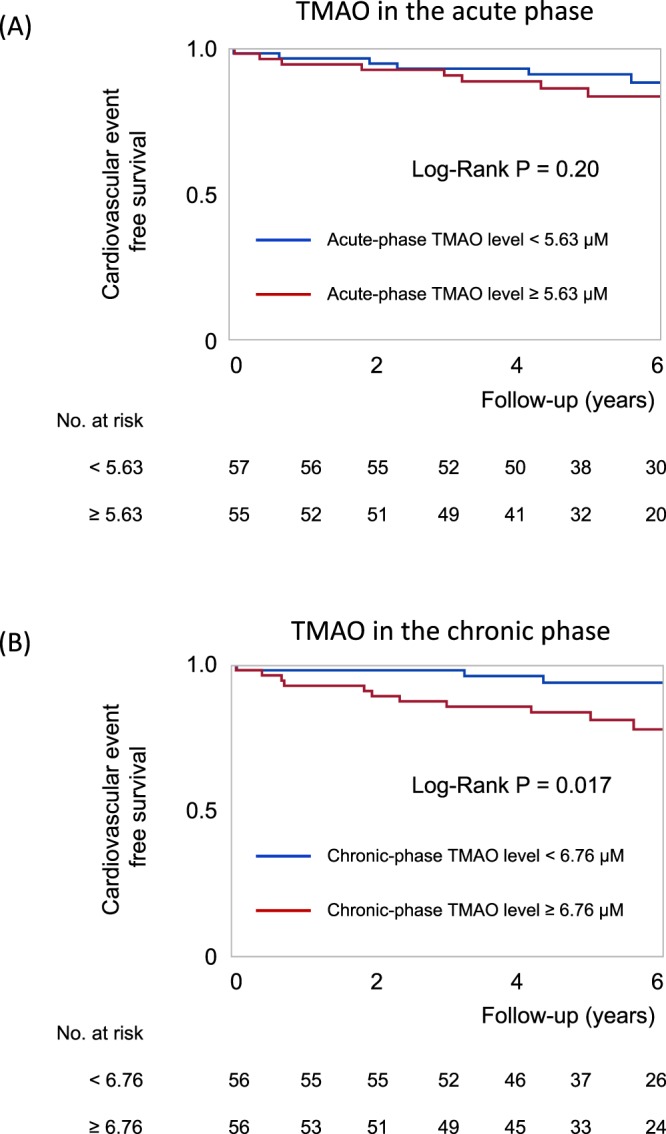
Kaplan-Meier curves for cardiovascular event-free survival after STEMI. (**A**) Patients were divided into two groups based on the median value (5.63 µM) of acute-phase TMAO levels. (**B**) Patients were divided into two groups based on the median value (6.76 µM) of chronic-phase TMAO levels.

Figure 3 shows the Kaplan-Meier curve indicating the cumulative event-free survival according to the acute-phase and chronic-phase TMAO levels. When STEMI patients were divided into two groups by the median value of acute-phase TMAO levels (5.63 µM), the event rate was not significantly different between the two groups (Fig. 3A). On the other hand, Fig. 3B shows that the time to cardiovascular events in the group with high chronic-phase TMAO levels (≥6.76 µM [median value]) was significantly shorter than that in patients with low chronic-phase TMAO levels (Log-Rank P = 0.017).

Univariate Cox proportional hazards analysis showed that a high concentration of TMAO in the chronic phase was a prognostic indicator of poor cardiovascular outcome after STEMI (hazard ratio (HR) for 0.1 increase in log chronic-phase TMAO levels: 1.226, 95% confidence interval (CI) 1.067 to 1.420, P = 0.004, Table 3); however, acute-phase TMAO levels were not significantly associated with events. Then, we performed a multivariate Cox proportional hazards model with adjustment for propensity scores, which demonstrated that high chronic-phase TMAO levels were independently associated with a high risk of cardiovascular events (adjusted HR for 0.1 increase in log chronic-phase TMAO levels: 1.343, 95% CI 1.122 to 1.636, P = 0.001, Table 3). The adjusted HR for patients with high chronic-phase TMAO (above median) compared to those with low values was 6.211 (95% CI 1.690 to 30.285, P = 0.005).

**Table 3:** Cox proportional hazard analysis for cardiovascular events.

Univariate analysis		Crude HR	95% CI	P	C-statistic
Multivariate analysis adjusted for propensity score		Adjusted HR	95% CI	P	
Acute phase	Log TMAO level, per 0.1	1.088	0.959 to 1.232	0.19	0.531
	TMAO ≥ median (5.63 μM)	1.875	0.711 to 5.236	0.20	
Chronic phase	Log TMAO level, per 0.1	1.226	1.067 to 1.420	0.004	0.738
	TMAO ≥ median (6.76 μM)	3.614	1.276 to 12.855	0.014	
Acute phase	Log TMAO level, per 0.1	1.078	0.947 to 1.228	0.25	
	TMAO ≥ median (5.63 μM)	1.715	0.625 to 4.922	0.29	
Chronic phase	Log TMAO level, per 0.1	1.343	1.122 to 1.636	0.001	
	TMAO ≥ median (6.76 μM)	6.211	1.690 to 30.285	0.005	

The propensity scores were estimated by fitting a logistic regression model with high TMAO levels in the acute or chronic phase (>median) as a dependent variable. The covariates included in the propensity score model were age, sex, hypertension, diabetes, dyslipidemia, smoking habits, systolic and diastolic blood pressure, triglycerides, high- and low-density lipoprotein cholesterol, glucose, hemoglobin A1c, estimated glomerular filtration rate, B-type natriuretic peptide, C-reactive protein, anterior myocardial infarction, atrial fibrillation, and medications on discharge (β-blockers, statins, angiotensin-converting enzyme inhibitors, and angiotensin II receptor blockers).

## Discussion

The major findings in the present study were as follows: (i) the plasma TMAO levels were significantly increased from the acute to chronic phase of STEMI, (ii) the higher chronic-phase TMAO levels were associated with coronary plaque progression, (iii) the chronic-phase TMAO level was a significant and independent predictor of future cardiovascular events in patients after STEMI, but acute-phase TMAO was not.

### TMAO, atherosclerosis, and cardiovascular disease

In 2011, TMAO was first identified using a targeted metabolomic approach aimed at identifying plasma metabolites, the levels of which predict the risk of cardiovascular diseases^4^. Dietary choline is metabolized to TMA by gut flora, and TMA (a gas) is then absorbed into the circulation and further metabolized to TMAO in the liver by the hepatic flavin monooxygenase family of enzymes. TMAO promotes cholesterol accumulation within macrophages and contributes to the development of atherosclerosis^4^. Recently, several studies have reported mechanistic links between TMAO and the pathogenesis of atherosclerosis and cardiometabolic diseases. Seldin *et al*. reported an *in vivo* study in which they demonstrated that TMAO acutely induces aortic endothelial cell inflammation through signaling of mitogen-activated protein kinase and nuclear factor-κB^8^. It is noteworthy that a higher TMAO level was associated with greater coronary plaque burden and higher plaque complexity as assessed by the SYNTAX score^11^. In addition to atherosclerotic plaque progression, platelet hyperreactivity plays an important role in the occurrence of vascular thrombotic complications, and TMAO directly increases platelet hyperreactivity and generates a prothrombotic phenotype *in vivo*, suggesting another potential pathway by which TMAO may contribute to thromboembolic events. Finally, Tang *et al*. reported the significant prognostic value of TMAO in predicting major adverse cardiac events, which was independent of traditional risk factors, C-reactive protein (CRP), and renal function^5^. As mentioned above, there is accumulating clinical and basic evidence suggesting a pathogenic role of TMAO in cardiovascular diseases. However, some studies have demonstrated no relationship between plasma TMAO levels and atherosclerotic vascular disease^12,13^. Further studies investigating the intracellular concentrations of TMAO, cellular signaling, and effects of TMAO on enzymes and other proteins are needed to establish the role of TMAO in human health and disease.

Plasma TMAO levels are affected by several factors, including kidney function, diet, hepatic flavin monooxygenase enzymes, and the gut microbiome^13–15^. Epidemiological, clinical and experimental studies have demonstrated that diet plays a central role in atherosclerotic cardiovascular diseases. Specific foods can be directly related to pro- or anti-inflammatory activity, and dietary patterns can be the major cause of many atherosclerotic risk factors^16^. Furthermore, diet is the most important cause of plasma TMAO variability^14^. Red meat, eggs, and dairy products contain precursors of TMA such as choline and carnitine. Smoking, which is considered to be another major cause of atherosclerotic cardiovascular diseases, is also suggested to have an effect on the intestinal microbiome and alter its composition^17,18^. Smoking may contribute to the development of intestinal and systemic diseases, at least partly through this interaction. Thus, plasma concentrations of TMAO exhibit wide variation within and between races and countries as well as individuals. A recent study of patients who were suspected of having acute coronary syndrome (ACS) reported that TMAO significantly predicted near-term and long-term cardiovascular outcomes^6^. This study included 530 patients from the Cleveland cohort and 1,683 patients from the Swiss ACS cohort, and the median value of TMAO was 2.87 µM (3.75 µM in the event group) in the Swiss ACS cohort and 4.28 µM (5.09 µM in the event group) in the Cleveland cohort. Another study of 720 patients with heart failure also reported the significant prognostic value of TMAO, and the level of TMAO in the population with heart failure (median value: 5.0 µM) was higher than those without heart failure (median value: 3.5 µM)^19^. The prognostic value of TMAO on cardiovascular outcomes was recently confirmed by a meta-analysis, which included 19 studies and 19,256 participants^20^. Among the studies included in this meta-analysis, the median values of TMAO levels were mostly from 3 to 7 µM. In the present study, the median value of TMAO was 5.63 µM in the acute phase and 6.76 µM in the chronic phase, which were comparable to previous studies from European countries and the United States. Based on the World Health Statistics of 2017, Japan was shown to be one of the healthiest and long-lived nations in the world (http://www.who.int/gho/publications/world_health_statistics/2017/en/), and the “Japanese diet” is an important factor. The Japanese diet is diverse and rich in rice and traditional foods, such as fish, vegetables, and soybeans, as well as meat, milk, oils, and fruits, which may decrease TMAO levels. This study population was composed of patients with STEMI, and thus TMAO levels in a Japanese general population have not yet been investigated. The levels of TMAO in Japanese individuals need to be further investigated and compared with those in other countries.

### TMAO as a residual risk factor and potential target in secondary prevention settings

In the present study, optimal secondary prevention therapies were performed with weight reduction and a high rate of adherence to evidence-based medications, as shown in the Results section. Nevertheless, plasma TMAO levels were significantly increased after these therapies, indicating that the current guideline-recommended therapies may not be effective in reducing TMAO. In the present study, chronic-phase plasma TMAO levels were strongly associated with cardiovascular events. This association may be partly explained by an absolute increase in plasma TMAO levels and a relative increase in clinical impact of TMAO on cardiovascular events derived from reduction of traditional risk by guideline-based secondary prevention therapy. Cardiovascular event rates have considerably declined by the current guidelines-based lipid lowering therapy with statin, blood pressure management, and lifestyle risk modifications considerably reduce patients’ risk for second cardiovascular events; however, long-term residual risk remains substantial. Thus, we suggest that reducing TMAO levels is an attractive measure to prevent cardiovascular events, especially in secondary prevention settings.

Several methods to reduce TMAO levels have been recently reported, such as diet intervention, probiotic/prebiotic/antibiotic intervention, and fecal microbiota transplantation^21^. Furthermore, it has been demonstrated that small-molecule antagonists inhibit microbial TMA formation, attenuating TMA and TMAO levels^22^. In addition, regulatory genetic factors affecting TMAO levels were also identified, which could be possible targets for therapeutic intervention^23^. These promising findings suggest that specific targeting of intestinal microbial TMA and TMAO production by specific inhibitors is feasible and could serve as a potential therapeutic approach to improve outcomes of those with cardiovascular diseases.

### Strengths and limitations

One of strengths of this study was the long follow-up period. Additionally, the investigators who collected event data were blinded to the TMAO and other clinical data, and no study patients were lost to follow-up. However, our study does have several limitations. First, the size of the study population is smaller than the required size calculated in our power analysis. In particular, the number of events is quite small. Since we enrolled only patients who underwent the assessment in both phases (acute and chronic), there may have been selection bias. Second, this study does not have a control group and thus cannot distinguish between spontaneous and treatment-induced developments. Third, smoking cessation, exercise, and diet therapy instructions were given to all patients; however, adherence to and achievement of these therapies were not assessed. Data on nutritional details were also not available. There is a possibility that the secondary preventive therapies used in this study population were not sufficient. Fourth, although implementation rates of secondary preventive medication were high, the percentages of patients who achieved target levels were not high enough: LDL cholesterol <100 mg/dl; 83%, LDL cholesterol <70 mg/dl; 24%, and triglycerides <150 mg/dl; 57%. Fifth, plasma samples were obtained in fasting conditions in the chronic phase, whereas in the acute phase, plasma samples were not necessarily obtained in fasting conditions. Therefore, in some patients admitted just after a meal, which included phosphatidylcholine, plasma TMAO levels might be increased^5^. Sixth, we assessed infarction sizes with peak levels of creatine kinase-myocardial band instead of cardiac magnetic resonance imaging and peak high-sensitivity troponin measurement. Seventh, because there were many missing data regarding left ventricular ejection fraction (LVEF) and left ventricular end-systolic volume (LVESV) on discharge and in the chronic phase, we could not include them in the analyses.

## Conclusions

Even after implementation of the current guideline-recommended secondary prevention therapy in STEMI patients, plasma TMAO levels were slightly increased from the acute phase to the chronic phase. The chronic-phase TMAO levels, which were assessed 10 months later, were associated with coronary plaque complexity and progression, as assessed by the SYNTAX score, and significantly predicted future cardiovascular events in patients after STEMI.

## Methods

**Figure 4:**
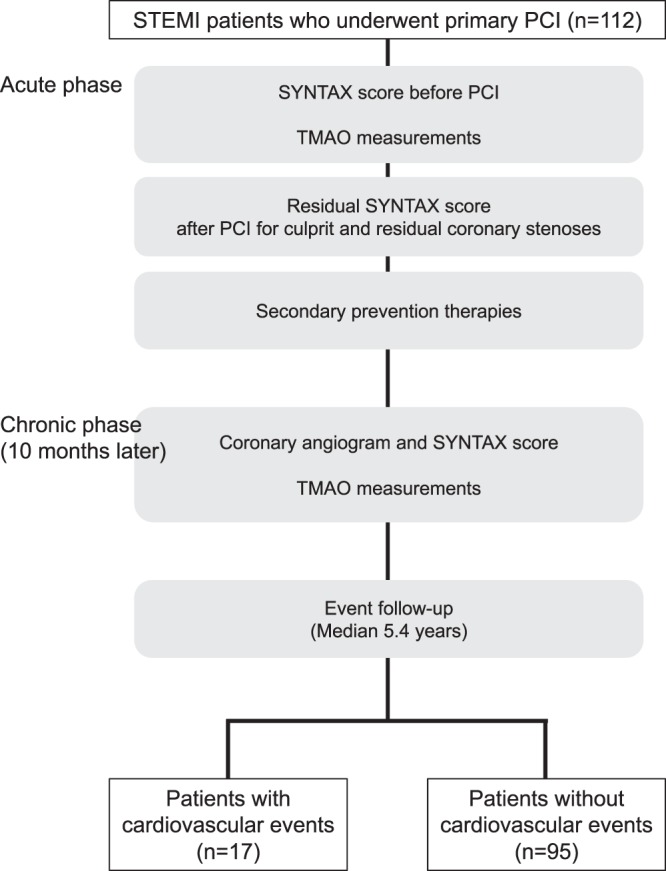
Study flow chart. PCI: percutaneous coronary intervention; STEMI: ST-segment elevation acute myocardial infarction; TMAO: trimethylamine N-oxide.

Figure 4 is a flow chart of the study. Plasma samples were collected from the study participants with STEMI at hospital admission before primary percutaneous coronary intervention and at the time of the follow-up coronary angiogram (10 months later). The guideline-based secondary prevention therapies were implemented in all patients. Plasma TMAO levels in the acute phase of STEMI and 10 months after implementation of secondary prevention therapies were analyzed using frozen samples. The primary outcome measure was the incidence of all-cause death, nonfatal myocardial infarction, nonfatal stroke, and unstable angina that required coronary revascularization. The secondary outcome measure was the progression of coronary atherosclerotic plaques as assessed by the SYNTAX score 10 months after the onset of STEMI. This study was performed according to the Declaration of Helsinki and approved by the Yokohama City University institutional review board. Written informed consent was obtained from all participants. The study is registered at http://www.clinicaltrials.gov (NCT03418285).

We enrolled 112 patients between January 2007 and January 2013 who were admitted to the Yokohama City University Medical Center with the diagnosis of their first STEMI and who underwent primary percutaneous coronary intervention within 12 h after onset and received follow-up coronary angiogram 10 months after the index intervention. The diagnosis of STEMI was determined by the presence of chest pain lasting more than 20 minutes in association with electrocardiographic changes (ST-segment elevation of ≥1 mV in at least two contiguous leads or new-onset left-bundle branch block) and increased creatine kinase or troponin.

### Quantification of TMAO in plasma samples

The plasma TMAO concentrations were quantified using liquid chromatography-mass spectrometry that employed a Q Exactive instrument (Thermo Scientific, San Jose, CA) equipped with a Dionex Ultimate 3000 UHPLC system (Thermo Scientific), and separation was carried out at 30 °C in an Xbridge amide column (4.6 × 150 mm, 3.5 mm, Waters, Milford, MA). The mobile phase consisted of a combination of 5% acetonitrile containing 5 mM ammonium acetate in MilliQ water (solution A) and 5 mM ammonium acetate in acetonitrile (solution B). The chromatogram was run under isocratic conditions at a flow rate of 0.7 mL/min, as follows: A/B = 95/5. A heated electrospray ionization (HESI) ion source was used for the ionization. The HESI parameters were optimized as follows: sheath gas flow rate 53 units, auxiliary gas unit flow rate 14, capillary temperature 269 °C, auxiliary gas heater temperature 45 °C, spray voltage 2500 V, and S lens RF level 55.

### Laboratory testing

We assessed the patients’ plasma lipid profiles (including LDL and HDL cholesterol and triglyceride levels); renal function, including plasma creatinine and estimated glomerular filtration rates (eGFRs); glucose; hemoglobin A1c; creatine kinase-myocardial band; B-type natriuretic peptide (BNP); and CRP. Creatine kinase-myocardial band was measured at 3-h intervals during the first 24 h after admission and then daily for the next 5 days, and the peak levels were determined.

### Secondary prevention therapies

Secondary prevention therapy was performed based on the Japan Atherosclerosis Society *Guidelines for the Prevention of Atherosclerotic Cardiovascular Diseases*. Printed educational information regarding secondary prevention, including smoking cessation, exercise, diet, and medical therapies, was provided for all patients. Dietary instructions were given by hospital dieticians during an individual 30-minute session, which included (i) total energy intake = ideal body weight × 125.6 kilojoules (30 kcal) (ideal body weight = height[m]^2^ × 22), which can be adjusted by physical activity. (ii) The recommended nutrient distribution of 50% to 60% carbohydrates, 15% to 20% proteins, 20% to 25% lipids, ≤300 mg/day cholesterol, ≥25 g/day dietary fiber, and ≤25 g/day alcohol. Exercise therapy instructions were given by the attending physicians as follows: (i) Exercise intensity was determined by the Karvonen method or the Borg scale (Karvonen method, target heart rate = [(220 - age) − resting heart rate] × 0.5 + resting heart rate; Borg scale, 11–13). (ii) Frequency: at least 30 minutes of exercise per day, 4 to 5 days per week.

### Follow-up and the definitions of cardiovascular events

After a follow-up coronary angiogram, cardiovascular events were followed in all patients. The primary efficacy outcome was a composite consisting of the first occurrence of nonfatal ischemic stroke, nonfatal myocardial infarction, unstable angina pectoris that required coronary revascularization, or all-cause death. Event follow-up was performed by phone call and medical record review, and three physicians on the event committee independently reviewed all medical records and death certificates to verify the diagnosis. In case of disagreement on an event classification, three reviewing physicians adjudicated the differences. The definition of myocardial infarction was a combination of symptoms, abnormalities on the electrocardiogram, and cardiac biomarker elevation. Ischemic stroke was defined as focal neurologic deficit with radiological evidence of brain infarction. Unstable angina pectoris was defined as new or accelerating myocardial ischemia symptoms accompanied by new ischemic ST-T-wave changes. In order to avoid observer bias, the physicians of the event committee were blinded to the clinical data and TMAO levels.

### SYNTAX score

Two interventional cardiologists (who were blinded to the TMAO results and clinical outcomes) evaluated the SYNTAX score using an online calculator (www.syntaxscore.com, version 2.28). These scores were calculated three times in all patients and included the acute-phase SYNTAX score, before any coronary interventions, residual SYNTAX score, at the time after interventions to all residual stenoses, including elective planned percutaneous coronary intervention, and the chronic phase SYNTAX score, which was measured 10 months after the onset of STEMI. In the absence of flow, the culprit lesions were scored as total occlusions of fewer than 3-month duration. The ΔSYNTAX score was defined as the chronic-phase SYNTAX – residual SYNTAX score, and plaque progression group was defined as the highest tertile of ΔSYNTAX score (≥3).

### Statistical analysis

All data analyses were performed using JMP Version 12.1.0 (12.0). The results are presented as a median (25th to 75th percentile) or n (%). Patients were divided into two groups according to the median value of acute-phase or chronic-phase plasma TMAO levels. Comparisons of groups at baseline were performed by a Wilcoxon test for continuous variables and Fisher’s exact test for categorical variables. We used the Wilcoxon signed-rank test to analyze differences in body weight, body mass index, hemoglobin A1c, LDL cholesterol, HDL cholesterol, triglycerides, and TMAO levels between the acute and chronic phases. To predict cardiovascular events, we calculated the area under the receiver operating characteristic curve for plasma TMAO and c-statistics. Propensity score adjustment was used to reduce the effect of bias and confounding in this observational study. The propensity scores were estimated by fitting a logistic regression model with high TMAO levels in the acute or chronic phase (>median) as a dependent variable. The covariates included in the propensity score model were age, sex, systolic and diastolic blood pressure, hypertension, diabetes, dyslipidemia, tobacco smoking, and levels of triglycerides, HDL and LDL cholesterol, glucose, hemoglobin A1c, eGFR, BNP, CRP, anterior myocardial infarction, atrial fibrillation, and medications on discharge. Medications included β-receptor blockers, statins, angiotensin-converting enzyme inhibitors, and angiotensin II receptor blockers. The Cox proportional hazard model was used to estimate the HR and 95% CI for the statistical impact of plasma TMAO levels on the clinical events. The HRs were expressed as risk increases (or decreases) per 0.1 increase in log TMAO levels. Event-free time was assessed by Kaplan-Meier curves using the log-rank test stratified by the median values of acute- and chronic-phase TMAO levels. Differences over time were assessed by the log-rank test. Patients who did not experience an event during follow-up were considered censored. The number of patients enrolled in this study (n = 112) was smaller than the required number estimated by power analysis (required sample size n = 132, α error 0.05, power 0.8, event rate 33%). A two-tailed P-value of <0.05 was considered statistically significant.

### Ethics approval and consent to participate

This study was performed according to the Declaration of Helsinki and approved by the institutional review board. Written informed consent was obtained from all participants.

## Data Availability

All authors have access to the database of this study.
